# CyDotian: a versatile toolkit for identification of intragenic repeat sequences

**DOI:** 10.1186/s43897-024-00113-3

**Published:** 2024-10-09

**Authors:** Huilong Chen, Gang Xu, Weina Ge, Shuyan Feng, Yanli Lin, Changqing Guo, Qianyi Jing, Xuekai Wang, Luiz Gustavo Nussio, Xiyin Wang, Fuyu Yang

**Affiliations:** 1 College of Grassland Science and Technology, China Agricultural University, Beijing, 100193 China; 2 College of Life Sciences, North China University of Science and Technology, Tangshan, 063210 Hebei China; 3 Department of Animal Sciences, Luiz de Queiroz College of Agriculture, University of Sao Paulo, Piracicaba, 13418-900 Brazil

Repetitive DNA sequences are highly dynamic components of the genome. Most repetitive sequences are located in intergenic regions, but some are also located in coding sequences (CDSs) or pseudogenes (Hartl 2000). The functions of repetitive sequences can be related to human genetic diseases, bacterial virulence, adaptive evolution, structural aberrations, transcriptional activity, and many other aspects (Van Belkum et al. 1998; Jin et al. 2004; Kashi and King 2006). The repetitive structure of DNA harbors many secrets yet to be discovered. The systematic study of repetitive DNA requires extensive algorithmic support.

There are already many tools that can be used to study repetitive sequences (Table S1). They are always designed to study genomic repeats and are not applicable to the study of all repeats within a gene, e.g., forward repeats, reversed repeats, perfect repeats, degenerate repeats, long repeats, and short repeats. The repeat-match program in the MUMmer toolkit (Kurtz et al. 2004) is a representative algorithm for identifying all repeats of a sequence. However, the repeats found by this method are perfect repeats, ignoring degenerate repeats, i.e., repeats with sequence differences. Another way to show all the repetitions within a sequence is a dot plot sequence comparison chart, such as the dotter tool (Sonnhammer and Durbin 1995). The drawback of this approach is that it is not possible to locate repeat positions and therefore does not provide statistical evidence about repeats within sequences.

In summary, to facilitate the study of intragenic repeats, we have written a tool (https://github.com/ChenHuilong1223/CyDotian) applicable to intragenic duplication research based on a dynamic programming algorithm for bridging the gap between the above two approaches and batch design for accelerating the utilization of the ever-increasing genetic resources. CyDotian can extract all the repeat sequences and support downstream analysis such as calculation of repeat density and depth maps (Fig. 1a, b).

**Fig. 1:**
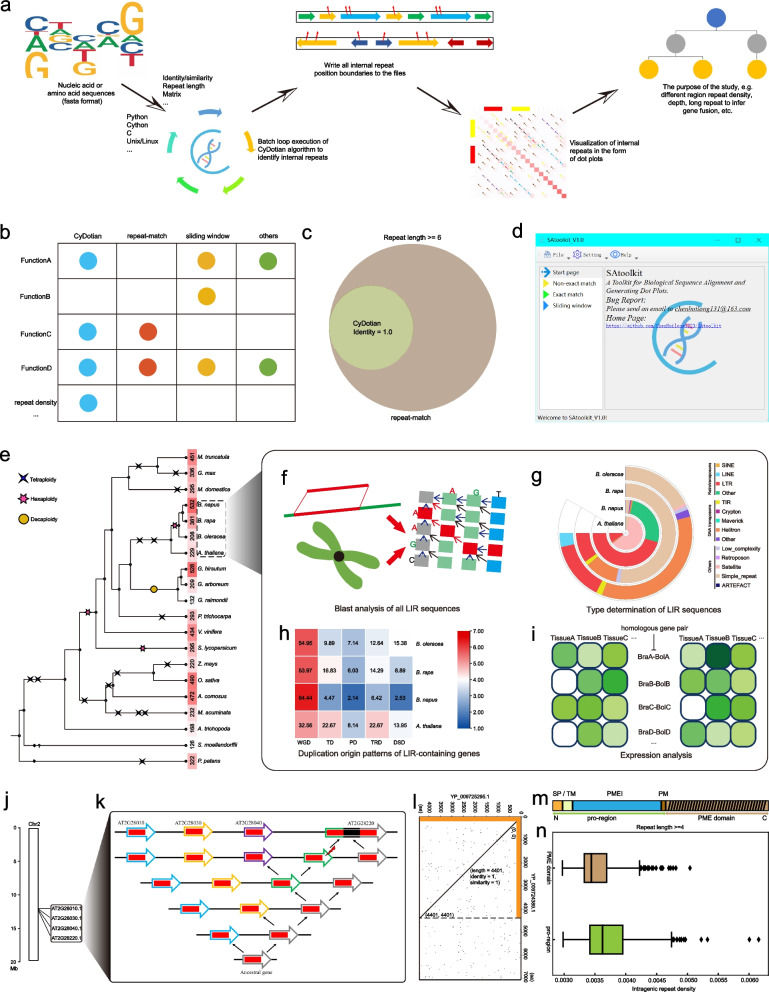
**a** Workflow of the CyDotian toolkit. **b** Comparison among CyDotian and other software or platforms. **c** Venn diagram of perfect repeat results identified by CyDotian versus perfect repeat results identified by repeat-match. **d** The starting screen of SAtoolkit, a toolkit that integrates three algorithms. **e** Evolutionary tree of life, paleopolyploidization, and number of LIR-containing genes in representative species of the plant kingdom, mainly horticultural plants. **f** Blast analysis of all LIR sequences in *Arabidopsis thaliana*, *Brassica napus*, *Brassica rapa*, and *Brassica oleracea*. **g** Statistical details of the number of element types that can be matched by all LIR in the four Cruciferae species. **h** Statistical details of the five duplication origins of the LIR-containing gene in four Cruciferae species. **i** Tissue expression of all LIR-containing and corresponding non-LIR-containing homologous gene pairs in *Brassica rapa* and *Brassica oleracea*. **j** Localization of the aspartate protease tandem repeat cluster gene containing *AT2G28220* on the chromosome. **k** An evolutionary model for tandem duplication and gene fusion of the *AT2G28220* gene in *Arabidopsis thaliana*. **l** Comparative dot plot results of two SARS-CoV-2 protein sequences. **m** Schematic diagram of the structure of the Type-1 PME amino acid. SP stands for signal peptide, TM stands for transmembrane domain, PMEI stands for pectin methylesterase inhibitor domain, PM stands for a processing motif (mainly RRLL or RKLL). **n** Comparison of the intragenic repeat density of the coding sequences of the pro-region and PME domain for all Type-1 PME genes in representative plants

We compared and benchmarked CyDotian with repeat-match and sliding window algorithms on 21 coding sequences derived primarily from horticultural plants (Figs. S1-22, Table S2, S3). The results showed that these 21 CDSs from different species containing long intragenic repeat (LIR) were evaluated consistently for all three algorithms. We found that the repeat pair identities of these sequences were all in the range of 0.45–1.0, and all of them had the highest number of repeat pairs in the interval of 0.65–0.7 and the lowest number of repeat pairs in the interval of 0.9–0.95. Moreover, the perfect repeats identified by CyDotian could all be included in the perfect repeat results identified via repeat-match (Fig. 1c), reflecting that CyDotian sacrifices some of the perfect repeats to find the degenerate repeats that repeat-match cannot find. More importantly, after comparing 21 intragenic repeat dot plots, we found that all three algorithms could correctly present the overall repeat sequence landscape within a gene via adjusting the parameter thresholds of different algorithms.

To allow the user to make the choice of algorithmic tools according to their preferences, we also batch the other two algorithmic implementations using our own programming logic (similar to the CyDotian toolkit implementation) and added them to the CyDotian toolkit. Moreover, for the convenience of researchers who are not good at using command line operations, we have developed an interactive visualization tool for CyDotian algorithm results-SAtoolkit (https://github.com/ChenHuilong1223/SAtoolkit) (Fig. 1d, S23).

To demonstrate the application potential of CyDotian, we utilized CyDotian to characterize the LIR of CDS in *Arabidopsis* and three *Brassica* species (Fig. 1e). Then, we blasted all the LIR sequences obtained above (Fig. 1f). We found that the same LIR sequences could match exactly across species or across genotypes (Table S4), suggesting that LIR sequences can span genomes.

We further analyzed the possible sources of these LIRs. We counted these LIR sequence types by blast comparison (identity = 100%) with all genomic component sequences in the TBGR database (http://www.tbgr.org.cn) (Fig. 1g, Table S5). The results showed that LIR sequences could be categorized into a wide variety of component types, such as transposon components, satellite, simple_repeat, and so on. Notably, most LIR sequences can match transposon elements.

By analyzing LIR-containing gene duplication origins, we found that all five duplication mechanisms [whole-genome duplication (WGD), tandem duplication (TD), proximal duplication (PD), transposed duplication (TRD), and dispersed duplication (DSD)] contributed to the origin of genes containing LIR. The highest number of genes originated from WGD, followed by TRD or TD and DSD, the fewest genes originated from PD (Fig. 1h, Table S6, S7).

We further explored the possible effects of LIR on gene expression. The results showed that the expression patterns of homologous genes containing and not containing LIR were variable and did not show a clear pattern (Fig. 1i, S24, Table S8). Therefore, the effect of LIR on gene expression is still an unanswered question, which needs to be investigated by specialized experiments in the future.

Interestingly, we found that the analysis of LIR also allowed us to infer whether the LIR host was a fusion gene. Here, we took the analysis of the LIR host gene *AT2G28220* of the *Arabidopsis* aspartate protease family as an example (Fig. 1j, k, S25). Firstly, the dot plot revealed that the length of the LIR was almost half of the whole sequence. By performing sequence analysis on the LIR sequence, we found that both the LIR and the preceding duplicated sequences had a complete feature structure, indicating that the LIR can be an independent gene sequence. By gene annotation correction, we excluded errors in gene prediction. Thus, this LIR host gene, *AT2G28220*, is a fusion gene in the genome of the *Arabidopsis* TAIR10 version. More deeply, combined with the fact that *AT2G28220* together with *AT2G28010*, *AT2G28030*, and *AT2G28040* form a tandem duplicate cluster, it therefore belongs to the tandem duplicated fusion gene type.

Due to the nature of the matrix algorithm, CyDotian can also perform comparisons between two different sequences and draw dot plots. Herein, we took the 12 protein sequences of SARS-CoV-2 as an example, and used CyDotian to discover sequence fragments that are repeated between these 12 protein sequences. In all two-by-two comparisons we found that YP_ 009725295.1 was part of the protein sequence belonging to YP_009724389.1 (identity = 100%) (Fig. 1l).

The Type-1 PME consists of an *N*-terminal pro-region and a *C*-terminal PME domain (Fig. 1m). The evolutionary analysis shows that the pro-region is more active and variable than the PME domain (Ge et al. 2022). We used CyDotian to compared the intragenic repeats of the two regions by the intragenic repeat density index $\rho$, we developed (Fig. 1n). The results showed that the CDS of the pro-region of the Type-1 PME gene in plants had a greater density of intragenic repeats than that of the PME domain and was statistically significant (*t*-test, *P*-value < 0.001), which suggests that this may be the driver of rapid pro-region evolution. Thus, we statistically demonstrate the nature of the Type-1 PME gene by CyDotian and show that the intragenic repeat density index $\rho$ can be an aspect for judging the nature of a gene.

Based on the above findings, we believe that CyDotian has high utility and significance for studying repeat sequences within genes and facilitating the utilization of large genetic resources.

## Supplementary Information

Supplementary Material 1. Materials and Methods.

Supplementary Material 2. Supplementary Figures 1-25.

Supplementary Material 3. Supplementary Tables 1-8.

Supplementary Material 4. Abbreviations.

## Data Availability

All raw data used can be found at the figshare site (https://doi.org/10.6084/m9.figshare.26176801). CyDotian is freely available for public use at GitHub (https://github.com/ChenHuilong1223/CyDotian). All scripts, default parameter files, and test data in the CyDotian toolkit are available on GitHub (https://github.com/ChenHuilong1223/CyDotian). Tutorials for installation and use are available at GitHub (https://github.com/ChenHuilong1223/CyDotian/wiki).
