# Supplementary material for: CyDotian: a versatile toolkit for identification of intragenic repeat sequences
Source: Mol Hortic. 2024 Oct 9;4:37. doi: 10.1186/s43897-024-00113-3 (PMC11462849; doi:10.1186/s43897-024-00113-3)
Supplement: Supplementary file 1 — Supplementary Material 1. Materials and Methods. [file 43897_2024_113_MOESM1_ESM.docx]

**CyDotian: a versatile toolkit for identification of intragenic repeat sequences**

Huilong Chen^1†^, Gang Xu^1†^, Weina Ge^2†^, Shuyan Feng^2^, Yanli Lin^1^, Changqing Guo^2^, Qianyi Jing^2^, Xuekai Wang^1^, Luiz Gustavo Nussio^3^, Xiyin Wang^2,*^, Fuyu Yang^1,*^

^1^College of Grassland Science and Technology, China Agricultural University, Beijing 100193, China

^2^College of Life Sciences, North China University of Science and Technology, Tangshan, Hebei 063210, China

^3^Department of Animal Sciences, Luiz de Queiroz College of Agriculture, University of Sao Paulo, Piracicaba 13418-900, Brazil

^†^Huilong Chen, Gang Xu and Weina Ge contributed equally to this work.

**^∗^Correspondence:**

Fuyu Yang: Email: yfuyu@126.com; Tel: 010-62733052; Fax: 010-62734252

Xiyin Wang: Email: wangxiyin@vip.sina.com

**Materials and Methods**

**Principle of the algorithm**

We developed the dynamic programming algorithm as follows:

Let the two sequences to be compared be $A=a_{1}a_{2}\ldots a_{n}, B=b_{1}b_{2}\ldots b_{m}$, where $n, m$ are the lengths of the sequences $A, B$ respectively.

Determine the substitution matrix.

Create a score matrix $H$ which has a size of $n+1$ rows and $m+1$ columns and initialize its first row and column, i.e.

$H_{k0}=H_{0l}=0 (0\leq k\leq n,0\leq l\leq m)$ (1)

where:

$H_{k0}$ - the first cell position on the first column of the score matrix $H$;

$H_{0l}$ - the first cell position in the first row of the score matrix $H$.

Scoring is done from left to right and top to bottom filling in the remainder of the score matrix $H$ with the following filling rules.

$H_{ij}=\max\left\{ \begin{aligned} &H_{i-1,j-1}+s\left( a_{i},b_{j} \right) (1\leq i\leq n,1\leq j\leq m) \\ &0 \end{aligned} \right.$ (2)

where:

$H_{i-1,j-1}$ -$a_{i}$, $b_{j}$ top left cell score;

$s(a_{j},b_{j})$ - the similarity score of the $a_{i}$, $b_{j}$ comparison;

$0$ - $a_{i}$, $b_{j}$ no similarity up to this point.

Backtracking: starting with the highest scoring element in the matrix $H$ (when there are multiple highest scores, priority is given to start with the cell where the later score is located) and backtracking to the previous position depending on the source of the score and so on until an element with a score of $0$ is encountered. The segment with the highest local similarity, i.e. the repeat, is produced in this process. A fragment with the second highest similarity can be obtained by backtracking from the highest scoring position outside the highest similarity backtracking process, i.e. after completing the first backtracking, backtracking from the highest scoring element outside the first backtracking region to obtain a second repeat. Similarly, a third repeat is found. This process is reiterated until all repetitions of greater than a certain length are found.

For identifying reversed repetition, one of the sequences in the horizontal or vertical position is reversed, and the sequence in the other position is kept unchanged. Then the algorithm steps described above are executed and the final output position is transformed to the correct position.

**Repeat density index**

To better reflect the repetition of different regions within the sequence, based on the consistency of the scoring matrix and the dot plot, we define a repeat density index $\rho$, with the following formula:

$\rho=\frac{q}{l^{2}}$ (3)

where:

$q$ - Total number of spatial backtracking paths for the scoring matrix;

$l$ - The length of the side of the square region of space (that is, the length of the region of the sequence).

The repeat density is the sum of the number of spatial backtracking paths in the sequence region under forward repetition condition plus the number of spatial backtracking paths in the sequence region under reverse repetition condition divided by the area of the region. The proposed repeat density index can well reflect the intragenic repetition of a sequence, and by comparing the size of the repeat density of different regions, the intragenic repetition of different regions can also be compared intuitively.

**Development of the toolkit**

To achieve an efficient output of the results, we used C to implement the algorithm and compiled it into an executable program. This executable program is then called in a batch. On the other hand, the downstream analysis tools are written in Python. These downstream analysis tools include processing the location and number of repeat segments, plotting dot plots, plotting depth plots, calculating repetition density, and outputting specific repeat segment comparison details. All are batch processed and exported, which is tremendously user-friendly. Users can suggest and optimize the development of all codes. For the development of GUI tools, we first compile the C language with Cython to generate Python modules. Then we use the PyQt5 library to create the GUI interface, and finally, we use Pyinstaller to package the Python program to generate a software installation package that can be distributed.

**Comparative analysis of individual genes and different tools in CyDotian applications**

Originally from the previous study (Wang et al. 2009), the CDS of maize C4 gene *U08401.1* (we named the file U08401.1.cds.fasta) was downloaded from the NCBI database (https://www.ncbi.nlm.nih.gov/search/all/?term=U08401.1) as an example of the application of the CyDotian algorithm tool. CyDotian and repeat-match tools were then used to identify *U08401.1* intragenic repetitive sequences, respectively, and Python scripts were written to count and visualize their results. To run repeat-match, we used the parameter command "repeat-match -f -n 6 U08401.1.cds.fasta". Next, we used Python scripts to visualize the dot plots of the overall repeat landscape within the *U08401.1* gene identified based on the CyDotian and repeat-match algorithms, respectively. Finally, we used the "sliding window" module of the self-written SAtoolkit software to visualize the dot plots of the overall repeat patterns within the *U08401.1* gene based on the sliding window algorithm. Finally, Adobe Illustrator (Ai) was used to stitch and retouch the above graphics.

To adequately evaluate the performance of the above three algorithms, we collected 20 representative species of the plant kingdom with complex genomic backgrounds, mainly horticultural plants (*Medicago truncatula*, *Glycine max*, *Malus domestica*, *Brassica napus*, *Brassica rapa*, *Brassica oleracea*, *Arabidopsis thaliana*, *Gossypium hirsutum*, *Gossypium arboreum*, *Gossypium raimondii*, *Populus trichocarpa*, *Vitis vinifera*, *Solanum lycopersicum*, *Zea mays*, *Oryza sativa*, *Ananas comosus*, *Musa acuminata*, *Amborella trichopoda*, *Selaginella moellendorffii*, and *Physcomitrella patens*), and recorded detailed information on the sources and versions of their genomic data (Table S2). Information on the evolutionary tree of life relationships between these species and the complex polyploidization history they have undergone were derived from Song et al. (Song et al. 2020). We employed TBtools to remove alternative splice sequences (Chen et al. 2020), then used CyDotian to batch identify coding sequences (CDSs) containing long intragenic repeat (LIR) (Here, we define a DNA sequence with a repeat length greater than or equal to 300 bp and an identity greater than or equal to 65% in a gene coding sequence as a LIR sequence.) in each species, and finally randomly selected a sequence from each species following the above process to fairly compare the performance of CyDotian, repeat-match, and sliding window algorithms.

To make a more comprehensive comparison of the functional nature of the software, we have collected as many relevant tools (BioAider (Zhou et al. 2020), CENSOR (Jurka et al. 1996), Dotlet (Junier and Pagni 2000), Dotter (Sonnhammer and Durbin 1995), EMBOSS (Dottup) (Rice et al. 2000), Gepard (Krumsiek et al. 2007), IRF (Das and Ghosh 2021), MUMmer (repeat-match) (Kurtz et al. 2004), and RepeatMasker (Chen 2004)) as possible that are currently in vogue, and compared them.

**Comparison and type annotation of all LIRs of four Cruciferae representative species**

To unravel the benefits of CyDotian's batch capabilities, all CDSs of *Arabidopsis* (Thale_cress-TAIR_10), *B. rapa* (Chiifu-v3.0), *B. oleracea* (JZS-v1.1), and [*B.*](https://www.ncbi.nlm.nih.gov/Taxonomy/Browser/wwwtax.cgi?lvl=0&amp;id=52824) *napus* (ZS11-v0.0) from the TBGR database (http://www.tbgr.org.cn) were downloaded (Liu et al. 2022). It is used for subsequent batch analysis. Then CyDotian was used to identify LIRs of four Cruciferae representative species in a batch (Parameters set in the configuration file CyDotian.config: fileType = 0, identityThr = 0.65, repeatLen = 300, DNA_Matrix = 0, mode = 0,1). Then, we used CyDotian to extract all the repeat sequences, the sequences of the LIRs in the four species were also self-compared using blastn (version 2.9.0+) (*e*-value ≤ 1*e*^-5^, identity = 100%, and length of the matched fragments ≥ 195bp) (Altschul et al. 1990), as well as compared with the full nucleic acid sequences of transposon elements (TEs) and others in the TBGR database, and data with identity = 100% were retained for subsequent analysis.

**Identification of the origin of duplication of all LIR-affiliated genes in four representative Cruciferae species**

To explore the origins of the genes that contain these LIRs, for genes containing LIRs in four Cruciferae representative species, we employed the diamond program (version 2.1.9) (Buchfink et al. 2021) and the DupGen_finder software (Qiao et al. 2019) to analyze them for duplication origin types. Referring to the previous study (Qiao et al. 2019), we used the *Nelumbo nucifera* genome as the reference genome (Wang et al. 2013). In order to ensure that each gene was assigned to a unique duplication type, we used the DupGen_finder-unique program to assign duplication types to each LIR-containing gene in the order WGD > TD > PD > TRD > DSD (Zhang et al. 2022). Genes for which the origin of duplication cannot be determined are denoted by unknown.

**Expression analysis**

To explore the possible effects of LIR on gene expression, we first identified all synteny homologous gene pairs between the two species, *B. rapa* and *B. oleracea*, using the JCVI software (Tang et al. 2024), and then extracted all gene pairs LIR-containing genes using Python scripts, followed by screening one gene containing LIR and another homologous gene pair without LIR as the subject of expression difference analysis. Finally, we manually obtained the expression values of each object gene from the BRAD database (http://brassicadb.cn/#/Transcriptome/) (Chen et al. 2021), which consisted of the transcripts per million (TPM) values of the gene in six tissues: callus, flower, leaf, root, silique, and stem. We used TBtools to visualize expression values as heatmaps and spliced and trimmed them with Ai.

**Methods and steps for inferring fusion genes via LIR**

Herein, we propose a method to infer fusion genes by identifying LIRs of CDSs via CyDotian. The principle is as follows: firstly, the set of coding genes containing LIRs is identified by CyDotian batch; then plotted as a dot plot for observation and extracted this LIR sequence; then the gene feature structure analysis of the LIR sequence determines that it is a sequence with a complete gene feature, and the structural annotations of the gene are corrected in order to exclude phenomena caused by genome annotation errors; and finally, in conjunction with the localization of the gene on the chromosome, we can determine whether it is a fusion gene and the possible mechanism of its formation (Fig. S25a).

For the fusion gene identification process the tools and steps used are as follows: based on the LIR host genes in *Arabidopsis*, we selected the *AT2G28220* gene, a member of the aspartate protease (AP) gene family. First, we collected sequences and structure annotation information of all gene members of the AP family from the TAIR database (https://www.arabidopsis.org/). All members were then analyzed for chromosomal localization using the homemade Python scripts, and the *AT2G28220* gene was identified to be grouped in a tandem repeat cluster. Then, the exon-intron structure of the gene was analyzed using the CFVisual software (Chen et al. 2022). Next, the amino acid sequences were then subjected to motif identification using the MEME website (Bailey et al. 2009) and functional structural domain identification using the NCBI-CDD (Marchler-Bauer et al. 2010), SMART (Letunic et al. 2021), and Pfam (Mistry et al. 2021) tools. The characteristic structure of a long repeat containing an intact AP gene member was subsequently identified. To exclude genomic annotation errors, gene structures were corrected using FGENESH software (Solovyev et al. 2006). Eventually, an evolutionary model of this tandem repeat gene cluster and fusion genes was deduced following our previous report (Wang et al. 2009). All of the data and code used during this period can be found at the figshare URL (https://doi.org/10.6084/m9.figshare.26176801).

**Comparative analysis of all amino acid coding sequences in the SARS-CoV-2 reference genome via CyDotian**

First, we downloaded 12 protein sequences of SARS-CoV-2 from NCBI (https://www.ncbi.nlm.nih.gov/labs/virus/vssi/#/virus?SeqType_s=Genome&VirusLineage_ss=taxid:2697049) and put them into the same file. Then, we used CyDotian to check the protein sequences for illegal characters. Next, CyDotian was used to identify the repeat sequences in 12 protein sequences of SARS-CoV-2 (parameters set in CyDotian.config: fileType = 1, similarityThr = 0.85, repeatLen = 12, aminoAcidMatrix = 1, mode = 0,1). Finally, the protein sequences in SARS-CoV-2 were self- and two-by-two compared using CyDotian, and the results were outputted to the folder "pairwise_comparison_result", in which "positions_original" is the position result of intra-proteome comparison, and "positions_original_dotplots" is the dotplot result of intra-proteome comparison.

**The comparative analysis of the intragenic repeat density of the pro-region and PME domains**

Identification of the CDSs of the PME genes containing the pro-region and the PME domain in 17 representative plants and the extraction of the CDSs of the two regions were achieved by programming, as described in the previous study (Ge et al. 2022). Then, the intragenic repetitive sequences of the CDS of these PMEs were analyzed using CyDotian (parameters set in CyDotian.config: fileType = 0, identityThr = 0.85, repeatLen = 4, DNA_Matrix = 1, mode = 0,1). Then CyDotian was used to identify the repeat density indices of all CDSs at once. Finally, a Python script was used to plot the box plots of the repeat densities of the pro-region and the PME domian, and the *t*-tests were calculated. The raw data used can be found at the figshare URL (https://doi.org/10.6084/m9.figshare.26176801).

**References**

Altschul SF, Gish W, Miller W, Myers EW, Lipman DJ. 1990. Basic local alignment search tool. *J Mol Biol* **215**: 403-410.

Bailey TL, Boden M, Buske FA, Frith M, Grant CE, Clementi L, Ren J, Li WW, Noble WS. 2009. MEME SUITE: tools for motif discovery and searching. *Nucleic Acids Res* **37**: W202-W208.

Buchfink B, Reuter K, Drost H-G. 2021. Sensitive protein alignments at tree-of-life scale using DIAMOND. *Nat Meth* **18**: 366-368.

Chen C, Chen H, Zhang Y, Thomas HR, Frank MH, He Y, Xia R. 2020. TBtools: an integrative toolkit developed for interactive analyses of big biological data. *Mol Plant* **13**: 1194-1202.

Chen H, Song X, Shang Q, Feng S, Ge W. 2022. CFVisual: an interactive desktop platform for drawing gene structure and protein architecture. *BMC Bioinformatics* **23**: 178.

Chen H, Wang T, He X, Cai X, Lin R, Liang J, Wu J, King G, Wang X. 2021. BRAD V3.0: an upgraded Brassicaceae database. *Nucleic Acids Res* **50**: D1432-D1441.

Chen N. 2004. Using Repeat Masker to identify repetitive elements in genomic sequences. *Current protocols in bioinformatics* **5**: 4.10. 11-14.10. 14.

Das G, Ghosh I. 2021. Benchmarking tools for DNA repeat identification in diverse genomes. *bioRxiv*: 2021.2009. 2010.459798.

Ge W, Chen H, Zhang Y, Feng S, Wang S, Shang Q, Wu M, Li Z, Zhang L, Guo H. 2022. Integrative genomics analysis of the ever-shrinking pectin methylesterase (PME) gene family in foxtail millet (Setaria italica). *Funct Plant Biol* **49**: 874-886.

Junier T, Pagni M. 2000. Dotlet: diagonal plots in a web browser. *Bioinformatics* **16**: 178-179.

Jurka J, Klonowski P, Dagman V, Pelton P. 1996. CENSOR—a program for identification and elimination of repetitive elements from DNA sequences. *Comput Chem* **20**: 119-121.

Krumsiek J, Arnold R, Rattei T. 2007. Gepard: a rapid and sensitive tool for creating dotplots on genome scale. *Bioinformatics* **23**: 1026-1028.

Kurtz S, Phillippy A, Delcher AL, Smoot M, Shumway M, Antonescu C, Salzberg SL. 2004. Versatile and open software for comparing large genomes. *Genome Biol* **5**: 1-9.

Letunic I, Khedkar S, Bork P. 2021. SMART: recent updates, new developments and status in 2020. *Nucleic Acids Res* **49**: D458-D460.

Liu Z, Li N, Yu T, Wang Z, Wang J, Ren J, He J, Huang Y, Shi K, Yang Q. 2022. The Brassicaceae genome resource (TBGR): A comprehensive genome platform for Brassicaceae plants. *Plant Physiol* **190**: 226-237.

Marchler-Bauer A, Lu S, Anderson JB, Chitsaz F, Derbyshire MK, DeWeese-Scott C, Fong JH, Geer LY, Geer RC, Gonzales NR. 2010. CDD: a Conserved Domain Database for the functional annotation of proteins. *Nucleic Acids Res* **39**: D225-D229.

Mistry J, Chuguransky S, Williams L, Qureshi M, Salazar GA, Sonnhammer EL, Tosatto SC, Paladin L, Raj S, Richardson LJ. 2021. Pfam: The protein families database in 2021. *Nucleic Acids Res* **49**: D412-D419.

Qiao X, Li Q, Yin H, Qi K, Li L, Wang R, Zhang S, Paterson AH. 2019. Gene duplication and evolution in recurring polyploidization–diploidization cycles in plants. *Genome Biol* **20**: 1-23.

Rice P, Longden I, Bleasby A. 2000. EMBOSS: the European molecular biology open software suite. *Trends Genet* **16**: 276-277.

Solovyev V, Kosarev P, Seledsov I, Vorobyev D. 2006. Automatic annotation of eukaryotic genes, pseudogenes and promoters. *Genome Biol* **7**: 1-12.

Song X-M, Wang J-P, Sun P-C, Ma X, Yang Q-H, Hu J-J, Sun S-R, Li Y-X, Yu J-G, Feng S-Y. 2020. Preferential gene retention increases the robustness of cold regulation in Brassicaceae and other plants after polyploidization. *Horticulture research* **7**.

Sonnhammer EL, Durbin R. 1995. A dot-matrix program with dynamic threshold control suited for genomic DNA and protein sequence analysis. *Gene* **167**: GC1-GC10.

Tang H, Krishnakumar V, Zeng X, Xu Z, Taranto A, Lomas JS, Zhang Y, Huang Y, Wang Y, Yim WC. 2024. JCVI: A versatile toolkit for comparative genomics analysis. *iMeta*: e211.

Wang X, Gowik U, Tang H, Bowers JE, Westhoff P, Paterson AH. 2009. Comparative genomic analysis of C4 photosynthetic pathway evolution in grasses. *Genome Biol* **10**: 1-18.

Wang Y, Fan G, Liu Y, Sun F, Shi C, Liu X, Peng J, Chen W, Huang X, Cheng S. 2013. The sacred lotus genome provides insights into the evolution of flowering plants. *The Plant Journal* **76**: 557-567.

Zhang Y, Zhang Y, Li B, Tan X, Zhu C, Wu T, Feng S, Yang Q, Shen S, Yu T. 2022. Polyploidy events shaped the expansion of transcription factors in Cucurbitaceae and exploitation of genes for tendril development. *Horticultural Plant Journal* **8**: 562-574.

Zhou Z-J, Qiu Y, Pu Y, Huang X, Ge X-Y. 2020. BioAider: an efficient tool for viral genome analysis and its application in tracing SARS-CoV-2 transmission. *Sustainable cities and society* **63**: 102466.
