# Supplementary material for: CyDotian: a versatile toolkit for identification of intragenic repeat sequences
Source: Mol Hortic. 2024 Oct 9;4:37. doi: 10.1186/s43897-024-00113-3 (PMC11462849; doi:10.1186/s43897-024-00113-3)
Supplement: Supplementary file 4 — Supplementary Material 4. Abbreviations. [file 43897_2024_113_MOESM4_ESM.docx]

Abbreviations

CDSs：Coding sequences

DSD：Dispersed duplication

LIR：Long intragenic repeat

PD：Proximal duplication

PME：Pectin methylesterase

SARS-CoV-2：Severe acute respiratory syndrome coronavirus 2

TD：Tandem duplication

TRD：Transposed duplication

WGD：Whole-genome duplication
