# Supplementary material for: CyDotian: a versatile toolkit for identification of intragenic repeat sequences
Source: Mol Hortic. 2024 Oct 9;4:37. doi: 10.1186/s43897-024-00113-3 (PMC11462849; doi:10.1186/s43897-024-00113-3)
Supplement: Supplementary file 2 — Supplementary Material 2. Supplementary Figures 1-25. [file 43897_2024_113_MOESM2_ESM.docx]

**CyDotian: a versatile toolkit for identification of intragenic repeat sequences**

Huilong Chen^1†^, Gang Xu^1†^, Weina Ge^2†^, Shuyan Feng^2^, Yanli Lin^1^, Changqing Guo^2^, Qianyi Jing^2^, Xuekai Wang^1^, Luiz Gustavo Nussio^3^, Xiyin Wang^2,*^, Fuyu Yang^1,*^

^1^College of Grassland Science and Technology, China Agricultural University, Beijing 100193, China

^2^College of Life Sciences, North China University of Science and Technology, Tangshan, Hebei 063210, China

^3^Department of Animal Sciences, Luiz de Queiroz College of Agriculture, University of Sao Paulo, Piracicaba 13418-900, Brazil

^†^Huilong Chen, Gang Xu and Weina Ge contributed equally to this work.

**^∗^Correspondence:**

Fuyu Yang: Email: yfuyu@126.com; Tel: 010-62733052; Fax: 010-62734252

Xiyin Wang: Email: wangxiyin@vip.sina.com

**Fig. S1.** Results of CyDotian analysis of the intragenic repeats of the coding sequence of the *Zea mays* C4 gene *U08401.1* and comparison with the results of repeat-match and sliding window algorithms. (a) Statistics of the number of all repetitive fragment pairs within the sequence identified by CyDotian at different identity intervals. (b) Venn diagram of perfect repeat results identified by CyDotian versus perfect repeat results identified by repeat-match. (c) Comparison of dot plots drawn from CyDotian, repeat-match's position results, and sliding window algorithm.

**Fig. S2.** Evolutionary tree of life, paleopolyploidization, and number of LIR-containing genes in representative species of the plant kingdom, mainly horticultural plants.

**Fig. S3.** Results of CyDotian analysis of the intragenic repeats of the coding sequence of the *Medicago truncatula* gene *Medtr2g068910.1* and comparison with the results of repeat-match and sliding window algorithms. (a) Statistics of the number of all repetitive fragment pairs within the sequence identified by CyDotian at different identity intervals. (b) Venn diagram of perfect repeat results identified by CyDotian versus perfect repeat results identified by repeat-match. (c) Comparison of dot plots drawn from CyDotian, repeat-match's position results, and sliding window algorithm.

**Fig. S4.** Results of CyDotian analysis of the intragenic repeats of the coding sequence of the *Glycine max* gene *Glyma.15G146400.2* and comparison with the results of repeat-match and sliding window algorithms. (a) Statistics of the number of all repetitive fragment pairs within the sequence identified by CyDotian at different identity intervals. (b) Venn diagram of perfect repeat results identified by CyDotian versus perfect repeat results identified by repeat-match. (c) Comparison of dot plots drawn from CyDotian, repeat-match's position results, and sliding window algorithm.

**Fig. S5.** Results of CyDotian analysis of the intragenic repeats of the coding sequence of the *Malus domestica* gene *MdoChr02g0072.1* and comparison with the results of repeat-match and sliding window algorithms. (a) Statistics of the number of all repetitive fragment pairs within the sequence identified by CyDotian at different identity intervals. (b) Venn diagram of perfect repeat results identified by CyDotian versus perfect repeat results identified by repeat-match. (c) Comparison of dot plots drawn from CyDotian, repeat-match's position results, and sliding window algorithm.

**Fig. S6.** Results of CyDotian analysis of the intragenic repeats of the coding sequence of the *Brassica napus* gene *BnaC02T0092800ZS* and comparison with the results of repeat-match and sliding window algorithms. (a) Statistics of the number of all repetitive fragment pairs within the sequence identified by CyDotian at different identity intervals. (b) Venn diagram of perfect repeat results identified by CyDotian versus perfect repeat results identified by repeat-match. (c) Comparison of dot plots drawn from CyDotian, repeat-match's position results, and sliding window algorithm.

**Fig. S7.** Results of CyDotian analysis of the intragenic repeats of the coding sequence of the *Brassica rapa* gene *BraA02g028880.3C* and comparison with the results of repeat-match and sliding window algorithms. (a) Statistics of the number of all repetitive fragment pairs within the sequence identified by CyDotian at different identity intervals. (b) Venn diagram of perfect repeat results identified by CyDotian versus perfect repeat results identified by repeat-match. (c) Comparison of dot plots drawn from CyDotian, repeat-match's position results, and sliding window algorithm.

**Fig. S8.** Results of CyDotian analysis of the intragenic repeats of the coding sequence of the *Brassica oleracea* gene *Bol004695* and comparison with the results of repeat-match and sliding window algorithms. (a) Statistics of the number of all repetitive fragment pairs within the sequence identified by CyDotian at different identity intervals. (b) Venn diagram of perfect repeat results identified by CyDotian versus perfect repeat results identified by repeat-match. (c) Comparison of dot plots drawn from CyDotian, repeat-match's position results, and sliding window algorithm.

**Fig. S9.** Results of CyDotian analysis of the intragenic repeats of the coding sequence of the *Arabidopsis thaliana* gene *AT1G33790.2* and comparison with the results of repeat-match and sliding window algorithms. (a) Statistics of the number of all repetitive fragment pairs within the sequence identified by CyDotian at different identity intervals. (b) Venn diagram of perfect repeat results identified by CyDotian versus perfect repeat results identified by repeat-match. (c) Comparison of dot plots drawn from CyDotian, repeat-match's position results, and sliding window algorithm.

**Fig. S10.** Results of CyDotian analysis of the intragenic repeats of the coding sequence of the *Gossypium hirsutum* gene *Gh_A01G1809* and comparison with the results of repeat-match and sliding window algorithms. (a) Statistics of the number of all repetitive fragment pairs within the sequence identified by CyDotian at different identity intervals. (b) Venn diagram of perfect repeat results identified by CyDotian versus perfect repeat results identified by repeat-match. (c) Comparison of dot plots drawn from CyDotian, repeat-match's position results, and sliding window algorithm.

**Fig. S11.** Results of CyDotian analysis of the intragenic repeats of the coding sequence of the *Gossypium arboreum* gene *Cotton_A_12477_BGI-A2_v1.0* and comparison with the results of repeat-match and sliding window algorithms. (a) Statistics of the number of all repetitive fragment pairs within the sequence identified by CyDotian at different identity intervals. (b) Venn diagram of perfect repeat results identified by CyDotian versus perfect repeat results identified by repeat-match. (c) Comparison of dot plots drawn from CyDotian, repeat-match's position results, and sliding window algorithm.

**Fig. S12.** Results of CyDotian analysis of the intragenic repeats of the coding sequence of the *Gossypium raimondii* gene *Gothu.00004553-RA* and comparison with the results of repeat-match and sliding window algorithms. (a) Statistics of the number of all repetitive fragment pairs within the sequence identified by CyDotian at different identity intervals. (b) Venn diagram of perfect repeat results identified by CyDotian versus perfect repeat results identified by repeat-match. (c) Comparison of dot plots drawn from CyDotian, repeat-match's position results, and sliding window algorithm.

**Fig. S13.** Results of CyDotian analysis of the intragenic repeats of the coding sequence of the *Populus trichocarpa* gene *Potri.018G049200.3* and comparison with the results of repeat-match and sliding window algorithms. (a) Statistics of the number of all repetitive fragment pairs within the sequence identified by CyDotian at different identity intervals. (b) Venn diagram of perfect repeat results identified by CyDotian versus perfect repeat results identified by repeat-match. (c) Comparison of dot plots drawn from CyDotian, repeat-match's position results, and sliding window algorithm.

**Fig. S14.** Results of CyDotian analysis of the intragenic repeats of the coding sequence of the *Vitis vinifera* gene *GSVIVT01000098001* and comparison with the results of repeat-match and sliding window algorithms. (a) Statistics of the number of all repetitive fragment pairs within the sequence identified by CyDotian at different identity intervals. (b) Venn diagram of perfect repeat results identified by CyDotian versus perfect repeat results identified by repeat-match. (c) Comparison of dot plots drawn from CyDotian, repeat-match's position results, and sliding window algorithm.

**Fig. S15.** Results of CyDotian analysis of the intragenic repeats of the coding sequence of the *Solanum lycopersicum* gene *Solyc08g008000.2.1* and comparison with the results of repeat-match and sliding window algorithms. (a) Statistics of the number of all repetitive fragment pairs within the sequence identified by CyDotian at different identity intervals. (b) Venn diagram of perfect repeat results identified by CyDotian versus perfect repeat results identified by repeat-match. (c) Comparison of dot plots drawn from CyDotian, repeat-match's position results, and sliding window algorithm.

**Fig. S16.** Results of CyDotian analysis of the intragenic repeats of the coding sequence of the *Zea mays* gene *Zm00001d002216_T001* and comparison with the results of repeat-match and sliding window algorithms. (a) Statistics of the number of all repetitive fragment pairs within the sequence identified by CyDotian at different identity intervals. (b) Venn diagram of perfect repeat results identified by CyDotian versus perfect repeat results identified by repeat-match. (c) Comparison of dot plots drawn from CyDotian, repeat-match's position results, and sliding window algorithm.

**Fig. S17.** Results of CyDotian analysis of the intragenic repeats of the coding sequence of the *Oryza sativa* gene *LOC_Os01g06380.1* and comparison with the results of repeat-match and sliding window algorithms. (a) Statistics of the number of all repetitive fragment pairs within the sequence identified by CyDotian at different identity intervals. (b) Venn diagram of perfect repeat results identified by CyDotian versus perfect repeat results identified by repeat-match. (c) Comparison of dot plots drawn from CyDotian, repeat-match's position results, and sliding window algorithm.

**Fig. S18.** Results of CyDotian analysis of the intragenic repeats of the coding sequence of the *Ananas comosus* gene *Aco000329.1* and comparison with the results of repeat-match and sliding window algorithms. (a) Statistics of the number of all repetitive fragment pairs within the sequence identified by CyDotian at different identity intervals. (b) Venn diagram of perfect repeat results identified by CyDotian versus perfect repeat results identified by repeat-match. (c) Comparison of dot plots drawn from CyDotian, repeat-match's position results, and sliding window algorithm.

**Fig. S19.** Results of CyDotian analysis of the intragenic repeats of the coding sequence of the *Musa acuminata* gene *Macma4_05_g04070.1* and comparison with the results of repeat-match and sliding window algorithms. (a) Statistics of the number of all repetitive fragment pairs within the sequence identified by CyDotian at different identity intervals. (b) Venn diagram of perfect repeat results identified by CyDotian versus perfect repeat results identified by repeat-match. (c) Comparison of dot plots drawn from CyDotian, repeat-match's position results, and sliding window algorithm.

**Fig. S20.** Results of CyDotian analysis of the intragenic repeats of the coding sequence of the *Amborella trichopoda* gene *evm_27.model.AmTr_v1.0_scaffold00071.19* and comparison with the results of repeat-match and sliding window algorithms. (a) Statistics of the number of all repetitive fragment pairs within the sequence identified by CyDotian at different identity intervals. (b) Venn diagram of perfect repeat results identified by CyDotian versus perfect repeat results identified by repeat-match. (c) Comparison of dot plots drawn from CyDotian, repeat-match's position results, and sliding window algorithm.

**Fig. S21.** Results of CyDotian analysis of the intragenic repeats of the coding sequence of the *Selaginella moellendorffii* gene *54187* and comparison with the results of repeat-match and sliding window algorithms. (a) Statistics of the number of all repetitive fragment pairs within the sequence identified by CyDotian at different identity intervals. (b) Venn diagram of perfect repeat results identified by CyDotian versus perfect repeat results identified by repeat-match. (c) Comparison of dot plots drawn from CyDotian, repeat-match's position results, and sliding window algorithm.

**Fig. S22.** Results of CyDotian analysis of the intragenic repeats of the coding sequence of the *Physcomitrella patens* gene *Pp6c26_7310V6.1* and comparison with the results of repeat-match and sliding window algorithms. (a) Statistics of the number of all repetitive fragment pairs within the sequence identified by CyDotian at different identity intervals. (b) Venn diagram of perfect repeat results identified by CyDotian versus perfect repeat results identified by repeat-match. (c) Comparison of dot plots drawn from CyDotian, repeat-match's position results, and sliding window algorithm.

**Fig. S23.** An introduction to the interface of SAtoolkit, a toolkit that integrates three algorithms. (a) The starting screen of SAtoolkit. (b) Interface for selecting scoring matrix parameters. (c) SAtoolkit's main interface. The left window is the software's control panel, and the right window is a user-interactable dot plot. (d) Zoom in on the schematic of the specified repeat region with the mouse, and the position marked by the crosshair cursor is the starting position of the forward repeat within that gene. (e) Statistical map of repeats within genes. (f) Statistical map of repeat depth within genes. (g) SAtoolkit's output screen. (h) ID input screen for comparison between different sequences.

**Fig. S24.** Tissue expression heatmap of all LIR-containing and corresponding non-LIR-containing homologous gene pairs in *Brassica rapa* and *Brassica oleracea*. Each row represents the tissue expression value (TPM) of the homologous gene pair. The name of the sample is on the horizontal axis and the gene ID is on the vertical axis. Yes means the gene is a LIR-containing gene and No means the gene does not contain LIR.

**Fig. S25.** The fusion gene *AT2G28220* was inferred based on CyDotian results. (a) Schematic diagram for inferring fusion genes with CyDotian based on long intragenic repeat recognition. (b) A dot plot of the intragenic repeats of long segments within the *AT2G28220* coding sequence is shown via CyDotian. (c) Comparison of the exon-intron structure of the aspartate protease tandem repeat cluster containing *AT2G28220*. (d) Comparison of the amino acid sequence structures encoded by the aspartate protease tandem repeat cluster gene containing *AT2G28220*.
